# Supplementary material for: Analysis of miR-29 Serum Levels in Patients with Neuroendocrine Tumors—Results from an Exploratory Study
Source: J Clin Med. 2020 Sep 6;9(9):2881. doi: 10.3390/jcm9092881 (PMC7565987; doi:10.3390/jcm9092881)
Supplement: Supplementary file 1 [file jcm-09-02881-s001.pdf]

Supplementary Material

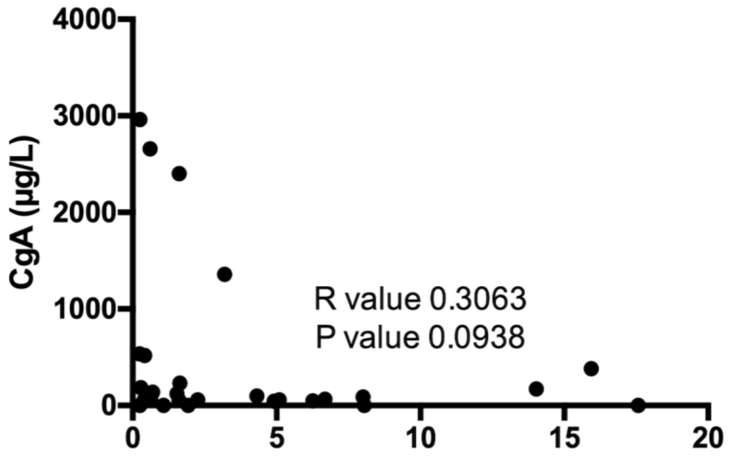

Supplementary Figure 1. Speerman rank analysis of chromogranin A and relative miR-29b expression.

Spearman rank analysis does not reveal a significant correlation between chromogranin A (CgA) values and relative miR-29b concentration in serum levels of NET patients (P = 0.0938, R = 0.3063)
